# A Dual Malnutrition Challenge in Tanzania Explored Through Logistic Regression Analysis

**DOI:** 10.3390/nu18081301

**Published:** 2026-04-20

**Authors:** Maryam Siddiqa, Gulzar H. Shah, Mahnoor Shahid Butt, Tehreem Asif, Bushra Shah

**Affiliations:** 1 Department of Mathematics & Statistics, International Islamic University, Islamabad 44000, Pakistan; maryam.siddiqa@iiu.edu.pk (M.S.); mahnoor.shahid.vt4146@iiu.edu.pk (M.S.B.);; 2 Department of Health Policy and Community Health, Jiann-Ping Hsu College of Public Health, Georgia Southern University, Statesboro, GA 30460, USA; bs06779@georgiasouthern.edu

**Keywords:** double-burden malnutrition (DBM), Composite Index Anthropometric Failure (CIAF), Tanzania

## Abstract

Background and Objectives: The double burden of malnutrition (DBM), characterized by the coexistence of malnutrition and overweight within the same household, has become a significant public health concern in low- and middle-income countries. Tanzania is undergoing a nutritional transition marked by persistent child malnutrition alongside increasing maternal overweight. This study examined socio-demographic, maternal, and child-level factors associated with DBM among children under five years in Tanzania. Methods: This cross-sectional study used data from the 2022 Tanzania Demographic and Health Survey, including a weighted sample of 5744 children under five and their mothers aged 15–49 years. DBM was defined as the presence of child malnutrition, measured using the Composite Index of Anthropometric Failure (CIAF), in households where the mother was overweight or obese. Bivariate chi-square tests and binary logistic regression analyses were conducted in STATA 17. Adjusted odds ratios (AORs) with 95% confidence intervals (CIs) were estimated to identify predictors of DBM. Results: DBM was more prevalent in rural areas. Significant predictors included birth order (AOR = 0.611, *p* = 0.030), child sex (AOR = 0.708, *p* = 0.011), perceived birth size (AOR = 0.270, *p* = 0.004), child age (AOR = 0.474, *p* < 0.001), maternal age (AOR = 0.599, *p* = 0.045), and maternal education (AOR = 0.604, *p* = 0.035). Higher maternal education reduced the likelihood of DBM, while firstborn male and small-sized children were at greater risk. Conclusions: DBM in Tanzania is influenced by both biological and socio-demographic factors. Integrated, multi-sectoral interventions targeting maternal education, prenatal care, and optimal maternal nutrition are essential to reduce DBM and achieve global malnutrition reduction targets.

## 1. Introduction

The double burden of malnutrition (DBM) denotes the concurrent contrasting presence of malnutrition (such as stunting, wasting, or micronutrient deficiencies) and overnutrition (including overweight, obesity, or diet-related infectious diseases) within individuals, households, and populations throughout the life course [1]. A severe double burden of malnutrition (DBM) has been formed at the individual, household, and population levels in many African countries due to a fast “nutrition transition” that has piled on an increasing burden of overweight and obesity on top of a persistently high burden of stunting, wasting, and deficiency in micronutrients. DBM is currently a common public health concern in low- and middle-income countries, particularly sub-Saharan Africa [2]. Child undernutrition remains a major issue in Southern Africa, with stunting rates ranging from 18% to 48%, and there is little research in the area. Key influences exist at the mother, child, family, and community levels, with maternal education and child birth weight being the most influential. Maternal age, stature, and employment are all factors that contribute to undernutrition, underscoring the need for diverse interventions [3].

According to global data, childhood obesity cases overtook underweight instances in 2025, with 9.4% of children aged 5 to 19 being overweight and 9.2% being underweight. Overweight affects around 38.9 million children under the age of five (approximately 5.6% prevalence based on WHO estimates), while stunting continues to impact approximately 22% of children under the age of five (~149 million). However, co-occurrence (overweight stunting) lacks specific global percentages; studies show increased dual burdens in low/middle-income countries [4,5]. Africa has the highest number of children who are stunted and wasted, and undernutrition is most common in low-income countries. Undernutrition negatively affects growth, morbidity, and mortality, and it has several adverse effects on physical and mental development [3]. The World Food Program estimates that as of 2022, approximately 350 million children are undernourished, 52 million children under five were wasted worldwide, of which 17 million were seriously wasted, and over 22.9% were stunted [6]. Malnutrition is a major public health issue, especially in Sub-Saharan Africa, where poverty is the root cause of over 90% of all nutritional disorders and two-thirds of undernutrition cases [7]. Multiple family members suffering from various types of malnutrition are referred to as DBM at the household level [8]. DBM at the household level varies by nation and is more common in lower–middle-income nations like Tanzania [9]. Four countries (Tanzania, Kenya, Madagascar, and Ethiopia) had anthropometric failure rates ranging from 24% to 44%, with children between the ages of 13 and 24 months being at greater risk. Reduced risks of undernutrition were associated with higher parental education, improved socioeconomic status, female sex, and postnatal checks. In Ethiopia and Tanzania, maternal education was important, as was paternal education in Kenya and Madagascar. Postnatal care and affluence also had protective effects [8,9,10].

The relationship between overweight moms and stunted children (SCOWT) is a paradox of the nutrition transition, in which changes in malnutrition patterns within the same household are brought about by economic progress [11]. DBM is often revealed to coexist in the same communities and even within the same families, for example, an overweight mother and a stunted or underweight child, in the regional analysis and national surveys [12]. The generational transfer of nutritional risk was highlighted by another study conducted in Ghana and Kenya, which indicated that maternal obesity and low birth weight were covariates of early childhood double-burden malnutrition [13]. DBM among children under five and its determinants, such as household poverty, low maternal education, and limited diet diversity, are frequently noted in pooled Demographic and Health Survey (DHS) analyses across 12 Eastern and Southern African countries [14]. Additionally, comparable trends are evident in Ethiopian household meta-analyses and South African national data [15]. Recent studies have highlighted the increasing prevalence of DBM in Africa. The pooled prevalence of DBM among mother–child pairs was 8.3% in a 2024 meta-analysis conducted in Ethiopia. There was notable variance by maternal education, wealth position, and place of residence [16]. Urbanization and economic inequality intensify the problem: food insecurity and micronutrient deficiencies persist, while energy-dense, nutrient-poor diets become more accessible, increasing the risk of malnutrition and diet-related noncommunicable illnesses throughout life [17]. Mother–child “dual burden” pairs, wealth- and education-related variations in DBM are documented in studies conducted in sub-Saharan African countries [15,16]. In Bahir Dar City, Northwest Ethiopia, 14.5% of mother–child pairs had the double burden of malnutrition [18]. As of 2018, 32% of children under five in Tanzania were stunted, making it one of the sub-Saharan African nations that suffer from malnutrition. Nonetheless, the prevalence rate remains above 40% in several areas, including Ruvuma, Iringa, Rukwa, Kigoma, Njombe, and Songwe. The stunting rate in Unguja North and Stone Town ranged from 20% to 24% [19]. Significant regional variance is seen in recent analyses based on Tanzania Demographic and Health Surveys, with some regions experiencing greater reductions in stunting [20].

Tanzania’s economy is expanding quickly, resulting in increased food supply. A shift in the economy that raises the average family income allows more households to buy more food, which may reduce malnutrition [21,22]. The prevalence of malnutrition among children under five in Tanzania, however, is still high and insufficient to meet the Sustainable Development Goals (SDGs) by 2030, despite slow improvements; for example, stunting decreased from roughly 50% in 1992 to nearly 30% in 2022, but about one in three children are still affected [20].

Moreover, the percentage of underweight children increased from 13.7% in 2014 to 14.6% in 2018 [22]. More than 30% of women in Tanzania aged 15 to 49 years are overweight and obese concurrently [16], which may be due to a reduction in physical activity at work and at home as well as an increase in the consumption of cheap and highly processed fast food and beverages [18]. DBM may rise in Tanzania due to the simultaneous effects of persisting malnutrition and growing obesity [23].

Although socioeconomic inequalities have been shown to underpin DBM in households, the relationship between household economic status and DBM is inconsistent [22,23]. Higher household economic status was associated with higher odds of DBM in poorer LMICs, while lower household economic status was associated with higher odds of DBM in wealthier LMICs [24].

The existing research on this topic in Tanzania has covered the mother–child double burden of malnutrition using nationally representative data. It has identified factors such as low birth weight, rural residence, and household wealth as important correlates [23]. Our study contributes to existing research in several ways. First, it defines child undernutrition using the Composite Index of Anthropometric Failure (CIAF), which captures overlapping anthropometric deficits rather than relying on a narrower phenotype. We also examine a broader set of maternal, child, and household characteristics within a single multivariable framework. In addition, our study provides updated national evidence for Tanzania, using the most recent DHS data. Thus, this study extends prior Tanzanian work by offering a more comprehensive and current assessment of household-level DBM among children under five. According to qualitative data, the main factors influencing women’s nutrition choices in rural areas of Tanzania, like Rorya, are poverty, food insecurity due to weather variations, low DBM awareness (being overweight is considered a status), and gender roles [24]. According to studies conducted in East and Southern African countries, child age, gender, mother’s BMI, income index, birth size, and sanitation access are all significantly associated with DBM among children under the age of five [25]. The prevalence of the double burden of malnutrition (DBM) in Tanzania was 5.6%, with substantial regional variation according to the Tanzania Demographic and Health Survey 2022. The strong interaction between family wealth and dietary variety showed that DBM was higher with increasing family wealth only among families that did not meet the minimum dietary diversity. This relationship was weakened and no longer significant in families that met dietary variety guidelines, highlighting the role of food quality in preventing DBM [26]. Perceived small birth size is related to increased DBM risk, indicating early-life obesity [25]. Being from rural areas and having a greater household income are strongly related to an increased risk of DBM in the mother–child pair condition, which is consistent with the dietary effects of transition in low-income countries [27].

The Tanzanian government has launched several programs and initiatives to address malnutrition and other health concerns. Among these activities are nutrition awareness programs like “Uzinduzi wa Kampeni ya Lishe Bora Kitaifa” in April 2022 and Campaign in the Box in November 2022. The midterm target of the National Multi-Sectoral Nutrition Action Plan 2016–2021, which aims to lower the prevalence of stunting from 34% to 32%, is another goal that the government has set for the nation to enhance people’s health [28]. To improve Tanzanians’ nutritional status, the government has also collaborated with several organizations, including the United Nations International Children’s Emergency Fund (UNICEF). To support Tanzania’s National Food and Nutrition Policy of 1992, UNICEF has been strengthening district nutritionists’ abilities [25,26]. Even with the government’s efforts, more needs to be done to address the nation’s nutritional issues. The report defines the double burden of malnutrition (DBM) as the co-occurrence of overweight or obesity and undernutrition (stunting/wasting) in the same population or household. It emphasizes how children’s inadequate diets, which include high-calorie but low-nutrient foods, cause both growth inadequacies and excessive weight gain [29]. Approximately 34% of children under five in the nation were stunted in 2020, which is more than the African region’s average (30.7%) [30]. Tanzania’s Nutritional Status for 2022 shows that 42% of children aged 0–5 are stunted [31].

This study addresses key gaps in the literature on child nutrition in Tanzania. Previous research has largely assessed undernutrition and overweight as separate conditions, with limited attention to their coexistence within the same household. Evidence on the double burden of malnutrition among children under five remains limited, particularly from nationally representative data. In addition, few studies have examined the combined role of maternal, child, and socioeconomic factors using recent DHS datasets. Thus, the need to address DBM in African countries requires a comprehensive approach that addresses obesogenic food environments and risks while promoting and supporting optimal diets and services early. In an effort to turn both sides of the malnutrition curve, there is a need for new estimates to combine nutrition-specific and nutrition-sensitive approaches with policies that can change food systems [32]. In light of economic changes, globalization exacerbates the double burden of malnutrition (DBM), which mostly affects the world’s poorest population. Targeted “double-duty” measures that address certain groups and lessen the disparate costs arising from international trade and societal changes are necessary to overcome this [33]. There is a grave need for accurate and data-informed assessments that can identify the factors associated with DBM in Tanzanian children under the age of five. This research aims to fill this need by comprehensively determining the factors that influence DBM in Tanzanian children under the age of five. This study aims to identify the factors contributing to the double burden of malnutrition among Tanzanian children under five, providing updated and comprehensive evidence to inform targeted interventions and policy planning.

## 2. Methods

### 2.1. Data Source and Study Design

**Figure 1:**
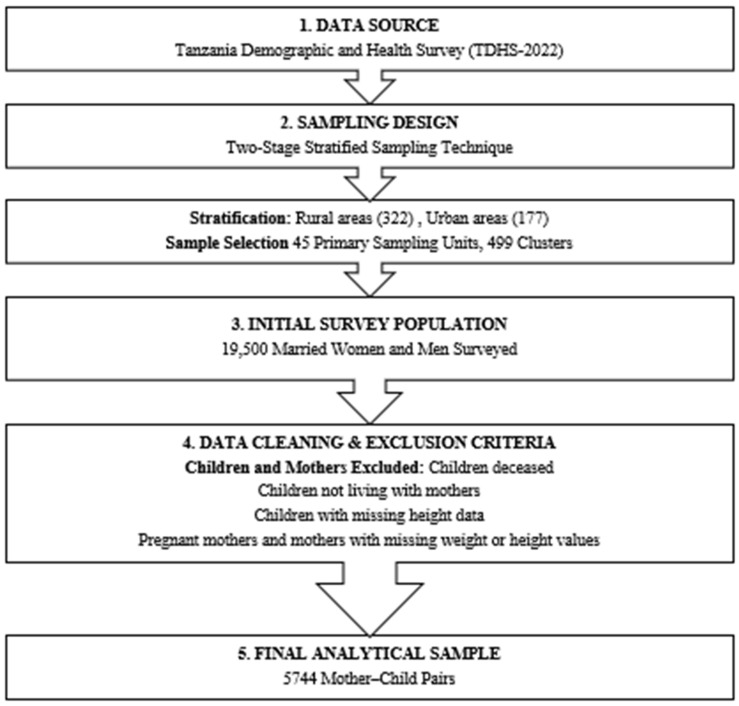
Flowchart of study population, sampling procedure, and data extraction.

This study’s data were taken from recent available Demographic and Health Surveys of Tanzania (TDHS-2022; TZKR82SV and TZIR82SV data file). The DHS is a survey conducted in more than 85 countries worldwide and is relatively nationally representative [32]. A two-stage stratified sampling technique was used to obtain data from the nationally representative Demographic and Health Survey (DHS). Rural and urban areas were stratified. A total of 45 primary sampling units and 499 clusters (322 urban and 177 rural) were chosen. A comprehensive list of households in each chosen cluster was compiled. For the study, 19,500 married women and men from various regions of the country were surveyed regarding the prevalence of double-burden malnutrition. Children who were deceased, were not living with their mothers, or had missing height data were excluded from the study. Moreover, pregnant mothers and those with missing weight or height values were also excluded. The determinants of DBM were examined using a final weighted sample of 5744 mother–child pairs as shown in Figure 1. In this study, the double burden of malnutrition (DBM) in the mother–child pair was defined as the coexistence of maternal overweight or obesity and child undernutrition within the same household [8,29]. A BMI ≥ 25 kg/m^2^ was used to identify maternal overweight or obesity [26]. Child undernutrition was quantified using the Composite Index of Anthropometric Failure (CIAF), which captures multiple forms of anthropometric failure. A z-score threshold below minus two standard deviations from the WHO reference population is used to define each of the three traditional anthropometric variables, which make up CIAF: height for age, weight for height, and weight for age [10]. If the mother was overweight and her child experienced anthropometric failure (CIAF), the DBM variable was coded 1; otherwise, it was coded 0.

### 2.2. Outcome Variable

The outcome variable is DBM: the number of overweight mothers with malnourished children in Tanzania.

### 2.3. Operational Definitions

Significant levels of both malnutrition and overnutrition coexist, which is known as the “double burden of malnutrition”. The coexistence of undernutrition and overweight/obesity in women living in the same residence is known as the “double burden of malnutrition in mother–child pairs”. Overweight: defined as having a body mass index (BMI) between 25 and 30 (kg/m^2^), and a BMI of 30 or higher is considered obese [34]. The children’s classification based on the Composite Index of Anthropometric Failure (CIAF) is shown in Table 1. Three types of malnutrition, wasting, stunting, and underweight, are used to categorize children. Children without anthropometric failure are represented by Group A. In contrast, various combinations of dietary deficiencies are represented by Groups B through Y. Rather than examining each sign independently, this classification provides a comprehensive assessment of total child anthropometric failure by capturing both single and multiple forms of malnutrition [35].

The Composite Index of Anthropometric Failure (CIAF), which combines several anthropometric deficiencies into a single indicator as shown in Table 1, is used in this study to quantify child malnutrition. Therefore, the coexistence of maternal overweight/obesity and child anthropometric failure within the same residence is known as the “double burden of malnutrition” at the mother–child level.

The Composite Index of Anthropometric Failure was used to estimate the overall burden of child undernutrition by capturing overlapping forms of anthropometric deficits not identified through individual indicators. This approach follows the classification framework proposed by Svedberg [36] and later refined by Nandy et al. [35]. CIAF integrates three standard anthropometric measures, height for age, weight for height, and weight for age, each defined by a z-score below minus two standard deviations from the World Health Organization reference population. Children were classified into seven mutually exclusive groups based on the presence and combination of these indicators. Group A includes children with no anthropometric failure. Group B represents wasting only. Group C includes wasting with underweight. Group D includes wasting, stunting, and underweight. Group E includes stunting with underweight. Group F represents stunting only. Group Y includes underweight only. Children classified in groups B through Y were considered undernourished, while group A served as the reference category. For regression analysis, CIAF was treated as a binary variable, with a value of 1 indicating the presence of any anthropometric failure and 0 indicating no failure.

CIAF provides a comprehensive measure by combining stunting, wasting, and underweight into a single index. This approach addresses limitations associated with separate indicators, which often fail to capture concurrent forms of malnutrition. Studies have applied CIAF to assess the full extent of undernutrition in low- and middle-income settings, where overlapping deficits are common [37,38]. This method improves the estimation of the total burden. It supports the identification of children with multiple failures, who face a higher risk of morbidity and mortality and are often missed in programs focused on single conditions. CIAF also supports analysis of environmental, maternal, and socioeconomic determinants that remain obscured when indicators are examined independently [39].

Data for the current study were drawn from the Tanzania Demographic and Health Survey (2022), and to construct the double burden of malnutrition variable, maternal BMI and child nutritional status were combined into a single binary outcome. Maternal BMI was classified into two groups. Mothers with BMI ≥ 25 kg/m^2^ were coded as 1, indicating overweight or obesity, while those with BMI < 25 kg/m^2^ were coded as 0. Child nutritional status was assessed using the Composite Index of Anthropometric Failure. Children with any form of anthropometric failure were coded as 1, while those without failure were coded as 0. These two indicators were then combined to define DBM. A value of 1 was assigned when a child with anthropometric failure lived with a mother who was overweight or obese. All other combinations were coded as 0. This approach captures the coexistence of undernutrition in the child and overnutrition in the mother within the same household, shown in Table 2.

### 2.4. Independent Variables

Independent variables were taken from the most recent DHS in Tanzania, including: place of residence (rural vs. urban), drinking water source (improved, unimproved), toilet facility (improved, unimproved), socioeconomic status (poor, middle, rich), father’s education (uneducated, educated), mother age (15–19, 20–24, 25–29, 30–34, 35–39, 40–44, 45–49), mother’s occupation (not working, working), maternal smoking (no, yes), intake of iron during pregnancy (no, yes), initiation of breastfeeding (immediately, within first hour, within first day), sex of child (male, female), child age in months (0–6, 7–12, 13–24, 25–36, 37–48, 49–60), birth order number (firstborn, 2nd–4th born, >5), baby postnatal checkup within two months (no, yes), milk consumption (no, yes), size of child at birth (small, average, large), and breastfed ever (no, yes).

### 2.5. Statistical Analysis

Descriptive statistics were used to describe the socio-demographic, child, and maternal characteristics of the study. We use Pearson $\varkappa^{2}$ test to examine if the magnitude of DBM varied by participant characteristics. Since DHS uses a multi-stage cluster sampling design, we used sample weights in all analyses. We used the weighting variable that DHS calculated. We employed the ‘svy’ command, and the sample weight (v005/1,000,000) was used to correct for over- and undersampling to improve the generalizability of our findings. The weights provide nationally representative estimates while accounting for the sampling strategy. The grouping and stratification were accounted for by using the proper survey design settings (svyset) in Stata to define the primary sampling unit (V021) and the stratification variable (V022) in the survey commands across all analyses, and by using survey-adjusted statistical techniques in Stata (Version 17; StataCorp LLC, College Station, TX, USA) [40]. In compliance with DHS analytical standards. We tested multicollinearity among independent variables using the correlation matrix and the Variance Inflation Factors (VIFs). Correlation coefficients below 0.80 and VIF values below 5 were considered indicative of the absence of concerning multicollinearity among the independent variables.

The association between the DBM and the explanatory variables of maternal, child, and socio-demographic characteristics was examined using a chi-square test of independence. Binary logistic regression was used to assess the risk factors for DBM. The strength of the association was measured using the Adjusted Odds Ratio (AOR), which is the ratio of the likelihood of the outcome in the exposed group to the likelihood in the unexposed group. To collect risk variables for the final model, a backward stepwise elimination technique was selected. “Stepwise backward elimination” is an iterative variable-selection method that starts with the full model and removes extraneous variables one at a time. At the initial step of fitting a multivariate model, all variables were included. Then, at each step, the variable with the highest *p*-value or the lowest F-statistic was removed, and the model-fitting process was repeated until only the variables significantly associated with the study’s result were selected and retained.

## 3. Results

Table 3 shows the proportion of DBM (double-burden malnutrition) for each independent variable. Among the 1376 respondents, 17.54%, who lived in rural areas, experienced double-burden malnutrition. A total of 850 individuals (19.25%) from households with a poor wealth index had suffered from double malnutrition. In terms of parental education, 19.31% of the 439 and 18.10% of the 261 mother–child pairs with no education had double malnutrition. Out of 959 viewers, 17.47% of male children had double malnutrition, which remains more prevalent in male children compared with female children. Around 17.50% of the households utilized unimproved toilet facilities. In the unadjusted descriptive analysis, DBM varied significantly by perceived birth size (*p* = 0.001), and the highest crude proportion was observed among children reported as large at birth. Child’s age in months, initiation of breastfeeding, place of residence, toilet facility, socioeconomic status, sex of the child, size of child at birth, maternal education, and father’s education were significantly associated with double burden of malnutrition (DBM) (*p* < 0.05). However, no significant association was found between DBM and factors such as source of drinking water, milk consumption, birth order number, baby postnatal checkup within 2 months, intake of iron during pregnancy, breastfed ever, maternal smoking, maternal age and mother’s occupation.

Table 4 displays a univariate logistic regression analysis based on DBM. Variables such as toilet facility, socioeconomic status, sex of child, size of child at birth, child’s age in months, mother’s education, and father’s education were found to be significantly associated with double-burden malnutrition (DBM) (*p*-values < 0.05).

These findings demonstrate that, in comparison to children of uneducated mothers, children of educated mothers are less likely to suffer from double-burden malnourishment (OR = 0.854; 95% C.I. 0.753–0.968). Double-burden malnutrition was less prevalent in children of educated fathers (OR = 0.663; 95% C.I. 0.552–0.797) than in children of uneducated fathers. These results also show that children aged 7–12 months (OR = 0.332; 95% C.I., (0.122–0.836)) had a lower risk of double malnutrition. Another variable that is associated with a reduced chance of double malnutrition is the size of the child at birth (OR = 0.442; 95% C.I. 0.176–0.769). (OR = 0.828; 95% C.I., 0.747–0.918) indicates that double malnutrition is less common in female children. Children from households with improved toilet facilities (OR = 0.872; 95% CI, 0.782–0.971) are less likely to suffer from double malnutrition. Children from rich households are less inclined to suffer from double malnutrition (OR = 0.591; 95% C.I., 0.524–0.665).

Table 5 presents a multivariate logistic regression analysis on DBM. DBM was significantly correlated with six variables: maternal age, mother’s education, child’s age in months, birth order, child’s sex, and child’s size at birth (*p*-values < 0.05).

Maternal age is associated with a lower risk of double malnutrition in children (AOR = 0.599; 95% C.I., 0.215–0.737) aged 25–29 years. According to these results, children aged 13–24 months (AOR = 0.474; 95% C.I., 0.237–0.827) had a lower risk of double malnutrition. Double malnutrition was less likely to occur in children who were larger at birth (AOR = 0.270; 95%, C.I., 0.162–0.695). The analysis suggests that double malnutrition is less prevalent in female children (AOR = 0.708; 95% C.I., 0.542–0.924). Children with a 2nd–4th birth order number (AOR = 0.611, 95% C.I., 0.391–0.945) had a lower risk of having DBM. The findings indicate that children of educated mothers (AOR = 0.664; 95% C.I., 0.459–0.960) are less inclined to experience double malnutrition than children of uneducated mothers.

**Figure 2:**
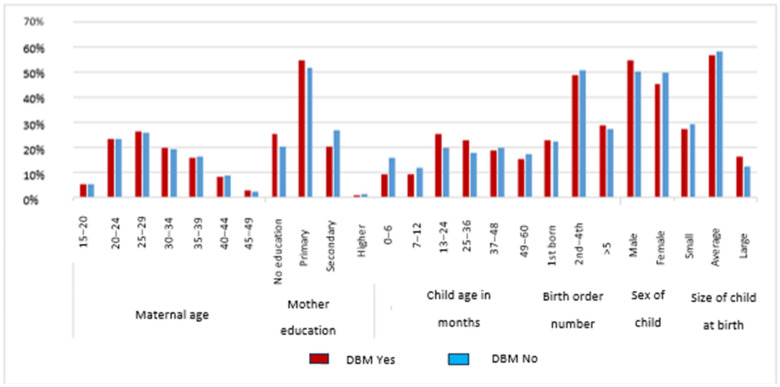
Prevalence of DBM by significant factors.

Figure 2 shows the prevalence of DBM (double burden of malnutrition) across various categories. “DBM Yes” is indicated by red bars. “DBM No” is shown by blue bars. Maternal age, mother’s education, child’s age in months, birth order number, sex of child, and size of child at birth are among the categories covered by the x-axis. Mothers between the ages of 25–29 had a slightly lower prevalence of DBM, but mothers 30 and older had a higher incidence. DBM proportions are higher in mothers who are very young (20–24) and elderly (40+). Mothers with no education had the highest DBM. DBM is lower for mothers with primary and secondary education. The 13–24-month group is less likely to have DBM. As the child becomes older than 36 months, it gradually increases. DBM is lower in birth orders 2nd–4th. Compared to male children, female children show higher levels of no DBM. DBM is lowest in large-sized children, moderate in average-sized children, and maximum in small-born children.

## 4. Discussion

Using nationally representative Tanzania data (TDHS-2022), this study investigated the factors contributing to the double burden of malnutrition (DBM) among children aged five and under. The results demonstrate that maternal- and child-related factors significantly influence the progression of DBM. This study underscores the critical challenge posed by DBM among children aged five in ESA countries. Birth order number, sex of child, size of child at birth, child age in months, maternal age, and maternal education are significantly associated with double-burden malnutrition. The co-occurrence of malnutrition and overweight calls for policies and programs to address the challenge of DBM through an integrated maternal and child health program by focusing on the identified factors. The findings of this study underscore the need to prioritize double-duty interventions that can simultaneously tackle multiple forms of malnutrition. This discussion highlights the consistency of these associations and the persistent need for targeted interventions in maternal health and early life to reduce DBM in Tanzania by situating our findings within the recent literature.

The concept of DBM, the negative effects of obesity and overweight, and other related health effects were not well understood by the participants. This is consistent with past research conducted in Tanzania with schoolchildren and their parents [41]. The lack of educational opportunities and the perception that being overweight or obese is a symbol of prestige and a pleasant life rather than a health issue may be the causes of Rorya district’s low level of DBM awareness [42]. The lack of information about the DBM is further complicated by the extensive nutritional change that is taking place in many low- and middle-income countries, including Tanzania. The term “nutritional transition” describes a change from conventional eating patterns to a diet that is lower in nutrients and higher in energy [24].

According to this study, the probabilities of DBM were significantly lower for children in the 2nd–4th birth order than for the firstborn [10]. Worldwide analyses conducted in Eastern and Southern Africa have found similar associations, indicating that firstborns are more susceptible to stunting and combined malnutrition outcomes than their later-born siblings [43]. Additionally, a Pakistani study found that firstborn children in households where the mother was already overweight were more likely to be malnourished, indicating a household-level DBM effect [44]. These findings indicated that this persistent disadvantage may be explained by early-life sensitivity in firstborns, eating habits, and parental inexperience. Although there was no statistically significant correlation between DBM and infants with a birth order larger than five in this study, this result should be regarded cautiously. The observed pattern suggests a non-linear association between birth order and DBM, with children of 2nd–4th birth order showing a significant association, while those of fifth or higher birth order did not. This may indicate that the effect of increasing birth order is not strictly monotonic and could be influenced by household resource allocation, maternal experience, or unmeasured contextual factors. Additionally, reduced precision in estimates for higher birth orders may have contributed to the lack of statistical significance.

According to the study conducted in Ethiopia, boys had a higher risk of DBM, which is in line with data from Asia and sub-Saharan Africa [45]. In Malawi, Namibia, and Zimbabwe, recent comparative research found that boys were more likely than girls to experience malnutrition and to live in circumstances that promote obesity [46]. Male gender is a persistent risk factor for the outcomes of child malnutrition. According to a scoping assessment of DBM in countries with low or middle incomes, the difference may be partially explained by biological factors such as boys’ greater metabolic needs and immunological sensitivity as well as gendered care behaviors in specific situations [47].

Size at birth was another major factor, with small- and average-sized children displaying increased DBM risk compared with those born large. Evidence from Ethiopia shows that children with low birth weights were consistently more likely to experience stunting and eventually become overweight [48]. The findings underline how restricted intrauterine growth exposes children to both poor early growth and long-term metabolic hazards, supporting the construct of developmental origins of health and illness [49].

Previous research also shows that children with low birth weights were consistently more likely to experience stunting and eventually become overweight [38]. It has also been demonstrated that maternal education in Tanzania affects the relationship between household wealth and the nutritional outcomes of children [40]. Furthermore, each of these results supports the idea that education is an integrated approach that improves mother and child nutrition over the long term.

The risk of DBM had a significant association with maternal age, with children of very young or elderly mothers being at higher risk. Global investigation conducted in sub-Saharan Africa reported that teenage mothers frequently lacked the nutritional reserves and capacity for parenting that are necessary to prevent malnutrition in their children [41]. According to regional studies conducted in Nigeria, poor child feeding outcomes were influenced by the obstetric risks and care limitations that older mothers encountered. These findings indicate that mother’s age influences a child’s susceptibility to DBM through both biological and social processes [42].

DBM was also associated with child age in months [43]. Infants 0–6 months old were more vulnerable than those 13–24 months old. Infants under six months old are frequently overlooked in nutrition initiatives, while having a significant incidence of undernutrition. Furthermore, socioeconomic studies conducted in East and Southern Africa showed that the prevalence of DBM is highest in the first six months of childhood and decreases as children switch to additional nutrition [44].

Other supplements such as iron supplementation during pregnancy are a key public health intervention due to its role in preventing maternal anemia and adverse birth outcomes. The World Health Organization recommends daily iron and folic acid supplementation (30–60 mg elemental iron) during pregnancy [50]. In the context of the double burden of malnutrition (DBM), maternal iron deficiency may coexist with overnutrition-related conditions, contributing to poor maternal and child health outcomes. Although other dietary supplements (e.g., folic acid, calcium, and multiple micronutrients) and medical interventions are also important [51], this study focused on iron intake due to its high relevance and the availability of consistent data in the TDHS 2022 dataset. Information on other supplements and drug use was either limited or not comprehensively captured. The lack of a significant association between iron intake and DBM in this study may reflect measurement limitations (e.g., self-reported intake), variability in adherence, and the stronger influence of socio-demographic and environmental factors. This does not diminish the importance of iron supplementation but highlights the multifactorial nature of DBM.

### 4.1. Strengths and Limitations

This study’s use of nationally representative data from the 2022 Tanzania Demographic and Health Survey, which improves the generalizability of the findings to Tanzanian children aged five and under, is one of the study’s strengths. Statistical strength and result accuracy are enhanced by its large sample size and consistent data collection. In light of the nutrition change affecting Eastern and Southern Africa, the study uses the double burden of malnutrition (DBM) approach, which offers an overall view of child nutrition by addressing both malnutrition and overweight. Additionally, it takes into account socio-demographic, mother and child variables such as maternal education, BMI, age, child sex, birth size, and socioeconomic position, supporting the idea that DBM is a complex problem impacted by a variety of factors. This study has some drawbacks that should be noted despite its advantages. First, it is difficult to establish causal links between the detected parameters and DBM because the TDHS data are cross-sectional. Second, the TDHS did not quantify the medications and dietary supplements individuals were taking, which could bias estimates of dietary micronutrient consumption. Moreover, even though this study emphasizes significant socioeconomic, child, and maternal variables, it does not fully account for health system factors that may also be important in determining DBM outcomes, such as the quality of prenatal care, food security, physical activity, and nutrition counseling. Lastly, although the ≥5th birth order category was adequately represented, the unequal distribution across birth order groups and reliance on firstborn children as the reference category may have affected estimate precision, potentially contributing to wider confidence intervals and non-significant results for higher birth orders.

### 4.2. Policy Implications

The results of this study highlight Tanzania’s double burden of malnutrition (DBM) and the urgent need for coordinated nutrition strategies by increasing maternal and child health services such as prenatal care, safe delivery procedures, newborn growth monitoring, and interventions that focus on early-life nutrition, especially for firstborns and low-birth-weight infants. Given that male children are more vulnerable, gender-sensitive treatments are essential and should include customized infant and young child feeding (IYCF) programs. Long-term benefits may result from expanding educational opportunities for women and integrating nutrition knowledge into educational programs. Furthermore, to guarantee long-lasting reductions in DBM across generations, policymakers must use a multi-sectoral strategy that connects the systems of social protection, agriculture, health, and education.

Our finding that higher maternal education is associated with improved child nutritional outcomes is supported by national nutrition initiatives in Tanzania that emphasize education and behavior changes as key strategies for improving maternal and child health. For example, the Improving Maternal Nutrition (IMAN) Project, implemented by United Nations Children’s Fund in collaboration with the Tanzania Food and Nutrition Center, aimed to strengthen health systems and promote uptake of maternal nutrition services including counseling and micronutrient supplementation during pregnancy [52]. This project contributed to evidence of national scale-up of enhanced antenatal nutrition interventions for pregnant women. Similarly, the Mwanzo Bora Nutrition Program (MBNP), a USAID-supported initiative implemented with local partners such as COUNSENUTH and Africare, used social and behavior change communication (SBCC) to improve nutrition practices at household and community levels, including support for maternal and child nutrition education [53,54]. These national efforts reflect the importance of maternal knowledge and empowerment closely linked to educational attainment as part of Tanzania’s broader nutrition policy environment and provide contextual support for our findings.

## 5. Conclusions

This study emphasizes that the coexistence of malnutrition and overweight is caused by a combination of biological and socio-demographic factors, highlighting the complex nature of the double burden of malnutrition (DBM) among Tanzanian children. The results indicate that DBM risk can be reduced by treatments that focus on equal child feeding practices, better prenatal care, and maternal education. Strategies that address malnutrition and rising overnutrition simultaneously must be integrated and context-specific to address DBM. Maternal and child health initiatives that enhance community-based nutritional education and enhance the socioeconomic circumstances of mothers and families need to be given top priority by policymakers. Countries like Tanzania can make significant progress toward achieving the Sustainable Development Goal (SDG) of eliminating all forms of malnutrition by putting these evidence-based strategies into practice. The findings indicated that maternal age, maternal education, child’s age in months, birth order, child’s sex, and size at birth are significant determinants of the double burden of malnutrition (DBM) in Tanzania. These findings suggest the need for a comprehensive policy framework that addresses both maternal and child factors influencing nutritional outcomes. Enhancing maternal education should be a central focus, as higher educational attainment and improved nutrition literacy among women are closely linked to better feeding practices and overall child wellbeing. Strengthening maternal and child health services, particularly by incorporating nutrition-focused counseling during antenatal and postnatal care, would help mitigate nutritional risks across maternal age groups and birth conditions. Additionally, policies that encourage proper infant and young child feeding practices, including exclusive breastfeeding, timely introduction of complementary foods, and consistent growth monitoring, are vital for reducing DBM prevalence. Given the role of birth order, integrating family planning with nutrition programs can promote healthier birth spacing and more equitable distribution of household resources. The observed relationship between the sex of the child and DBM further highlights the importance of designing gender-responsive nutrition and health interventions to ensure equitable access for both boys and girls.

The regression results support targeted planning based on identified risk patterns. Children of mothers aged 25 to 29 years show lower odds of DBM, which indicates the need to focus on providing support to younger and older maternal age groups. Maternal education shows a protective effect, which supports investment in female education and nutrition literacy programs. Child-level factors also guide intervention timing. Children aged 13 to 24 months have a reduced risk of DBM, which highlights the importance of early-life nutrition before this age period. Larger birth size is associated with lower risk, which supports strengthening prenatal care and maternal nutrition. Female children show lower odds, which indicates the need for equitable strategies that address risk among male children. Birth order also shows a measurable effect, with second- to fourthborns linked to a lower risk, which supports integrating family planning with nutrition programs. These effect sizes provide a basis for prioritizing high-risk groups and improving the efficiency of intervention planning for national nutrition strategies.

These findings support a coordinated policy approach. Priority should be given to maternal education, early child feeding practices, prenatal and postnatal care, and family planning services. Community-based nutrition education and improved socioeconomic conditions for mothers and families remain central to reduce DBM. The use of model-based effect estimates helps guide resource allocation to high-risk groups and improves the efficiency of intervention planning in Tanzania.

## Figures and Tables

**Table 1:** Categorization of child anthropometric failure based on CIAF components (wasting, stunting, and underweight).

Group	CIAF	Wasting	Stunting	Underweight
A	No failure	No	No	No
Anthropometric Failure group				
B	Wasting only	Yes	No	No
C	Wasting and underweight	Yes	No	Yes
D	Wasting, underweight, and stunting	Yes	Yes	Yes
E	Stunting and underweight	No	Yes	Yes
F	Stunting only	No	Yes	No
Y	Underweight only	No	No	Yes

**Table 2:** Construction of double-burden malnutrition (DBM).

Variable	Indicator/Cut-Off	Coding	Description
Maternal BMI	BMI ≥ 25 kg/m^2^	1	Overweight/Obese
	BMI < 25 kg/m^2^	0	Not Overweight/Obese
Child Anthropometric Failure (CIAF)	Any Anthropometric Failure group	1	Yes (Anthropometric Failure Present)
	No anthropometric failure	0	No (Anthropometric Failure Absent)
Double Burden of Malnutrition (DBM)	Mother (BMI ≥ 25) and Child (CIAF = 1)	1	Yes (DBM Present)
	Otherwise	0	No (DBM Absent)

**Table 3:** Proportion of DBM for each independent variable in Tanzania (row percentages).

Characteristics	Tanzania (TDHS-2022)		
Double-burden malnutrition (DBM)	No DBM Frequency (%)	Yes DBM Frequency (%)	$\varkappa^{2}$/*p*-values
Societal Characteristics			
Place of residence			36.910/**<0.001**
Urban	2565 (87.30)	373 (12.70)	
Rural	6469 (82.46)	1376 (17.54)	
Source of drinking water			0.084/0.772
Unimproved	7261 (83.73)	1411 (16.27)	
Improved	1773 (83.99)	338 (16.01)	
Toilet facility			6.197/**0.013**
Unimproved	2876 (82.50)	610 (17.50)	
Improved	6158 (84.39)	1139 (15.61)	
Socioeconomic status			77.771/**<0.001**
Poor	3566 (80.75)	850 (19.25)	
Middle	1835 (82.58)	387 (17.42)	
Rich	3633 (87.65)	512 (12.35)	
Child Characteristics			
Milk consumption			1.918/0.166
No	5146 (83.21)	1038 (16.79)	
Yes	412 (85.65)	69 (14.35)	
Initiation of breastfeeding			6.751/**0.034**
Immediately	4758 (82.89)	982 (17.11)	
Within 1st hour	1156 (85.06)	203 (14.94)	
Within 1st day	48 (92.31)	4 (7.69)	
Birth order number			2.748/0.253
1st born	2017 (83.59)	396 (16.41)	
2nd–4th	4571 (84.32)	850 (15.68)	
>5	2446 (82.94)	503 (17.06)	
Sex of child			12.882/**<0.001**
Male	4530 (82.53)	959 (17.47)	
Female	4504 (85.08)	790 (14.92)	
Size of child at birth			13.976/**0.001**
Small	1598 (83.93)	306 (16.07)	
Average	3179 (83.22)	641 (16.78)	
Large	666 (78.35)	184 (21.65)	
Baby postnatal checkup within 2 months			1.248/0.264
No	1314 (82.59)	277 (17.41)	
Yes	2646 (83.87)	509 (16.13)	
Child’s age in months			75.855/**<0.001**
0–6	507 (77.52)	147 (22.48)	
7–12	378 (72.14)	146 (27.86)	
13–24	650 (61.03)	415 (38.97)	
25–36	579 (60.76)	374 (39.24)	
37–48	604 (65.30)	321 (34.70)	
49–60	567 (69.66)	247 (30.34)	
Parental Characteristics			
Breastfed ever			0.632/0.426
No	4307 (83.49)	852 (16.51)	
Yes	4727 (84.05)	897 (15.95)	
Maternal smoking			0.422/0.516
No	9002 (83.79)	1741 (16.21)	
Yes	32 (80.00)	8 (20.00)	
Intake of iron during Pregnancy			2.420/0.120
No	845 (81.64)	190 (18.36)	
Yes	4006 (83.63)	784 (16.37)	
Maternal age			2.697/0.846
15–19	438 (83.27)	88 (16.73)	
20–24	2079 (83.56)	409 (16.44)	
25–29	2378 (84.24)	445 (15.76)	
30–34	1793 (84.14)	338 (15.86)	
35–39	1391 (83.04)	284 (16.96)	
40–44	723 (83.10)	147 (16.90)	
45–49	232 (85.93)	38 (14.07)	
Mother’s education			55.882/**<0.001**
No education	1835 (80.69)	439 (19.31)	
Primary	4667 (83.03)	954 (16.97)	
Secondary	2424 (87.35)	351 (12.65)	
Higher	108 (95.58)	5 (4.42)	
Mother’s occupation			0.482/0.487
Not working	3063 (84.13)	578 (15.87)	
Working	5971 (83.60)	1171 (16.40)	
Father’s education			46.078/**<0.001**
No education	1181 (81.90)	261 (18.10)	
Primary	4205 (82.06)	919 (17.94)	
Secondary	1923 (87.21)	282 (12.79)	
Higher	233 (92.09)	20 (7.91)	

**Table 4:** Univariate logistic regression analysis on double-burden malnutrition in Tanzania (TDHS-2022).

Variables	Categories	Tanzania (TDHS-2022) Overall Model		
		OR	C.I	*p*-values
Societal Characteristics				
Place of residence	Urban *	-	-	-
	Rural	1.462	(1.293–1.654)	<0.001
Source of drinking water	Unimproved *	-	-	-
	Improved	0.981	(0.861–1.116)	(0.772)
Toilet facility	Unimproved *	-	-	-
	Improved	0.872	(0.782–0.971)	**(0.013)**
Socioeconomic status	Poor *	-	-	-
	Middle	0.884)	(0.774–1.010)	(0.071)
	Rich	0.591	(0.524–0.665)	**(<0.001)**
Child Characteristics				
Milk consumption	No *	-	-	-
	Yes	0.830	(0.637–1.080)	(0.167)
Initiation of breastfeeding	Immediately *	-		-
	Within 1st hour	0.850	(0.721–1.002)	(0.054)
	Within 1st day	0.403	(0.145–1.122)	(0.082)
Birth order number	1st born *	-	-	-
	2nd-4th	0.947	(0.831–1.078	(0.414)
	>5	1.047	(0.906–1.209)	(0.529)
Sex of child	Male *	-	-	-
	Female	0.828	(0.747–0.918)	**(<0.001)**
Size of child at birth	Small *	-	-	-
	Average	1.052	(0.907–1.222)	(0.497)
	Large	0.442	(0.176–0.769)	**(<0.001)**
Baby postnatal checkup within 2 months	No *	-	-	-
	Yes	0.912	(0.777–1.071)	(0.264)
Child’s age in months	0–6 *	-	-	-
	7–12	0.332	(0.122–0.836)	**(0.034)**
	13–24	2.202	(1.76–2.746)	(<0.001)
	25–36	2.227	(1.779–2.789)	(<0.001)
	37–48	1.832	(1.459–2.302)	(<0.001)
	49–60	1.502	(1.185–1.903)	(0.001)
Parental Characteristics				
Breastfed ever	No *	-	-	-
	Yes	0.959	(0.865–1.062)	(0.426)
Maternal smoking	No *	-	-	-
	Yes	1.292	(0.594–2.809)	(0.517)
Intake of iron during Pregnancy	No *	-	-	-
	Yes	0.870	(0.730–1.036)	(0.120)
Maternal age	15–19 *	-	-	-
	20–24	0.979	(0.760–1.260)	(0.870)
	25–29	0.931	(0.725–1.196)	(0.578)
	30–34	0.938	(0.725–1.212)	(0.627)
	35–39	1.016	(0.781–1.320)	(0.904)
	40–44	1.011	(0.757–1.351)	(0.936)
	45–49	0.815	(0.539–1.231)	(0.332)
Mother’s education	No education *	-	-	-
	Primary	0.854	(0.753–0.968)	**(0.014)**
	Secondary	0.605	(0.519–0.705)	**(<0.001)**
	Higher	0.193	(0.0784–0.477)	**(<0.001)**
Mother’s occupation	Not working *	-	-	-
	Working	1.039	(0.932–1.158)	(0.487)
Father’s education	No education *	-		
	Primary	0.988	(0.849–1.151)	(0.886)
	Secondary	0.663	(0.552–0.797)	**(<0.001)**
	Higher	0.388	(0.241–0.625)	**(<0.001)**

Note: OR = adjusted odd ratio, C.I = confidence interval, bolded = significant *p*-values, * shows reference category.

**Table 5:** Binary logistic regression analysis on double-burden malnutrition in Tanzania.

**Variables**	**Categories**	**Tanzania (TDHS-2022)** **Overall Model**		
		AOR	C.I	*p*-values
Socio-demographic Characteristics				
Place of residence	Urban *	-	-	-
	Rural	1.268	(0.876–1.836)	(0.207)
Source of drinking water	Unimproved *	-	-	-
	Improved	0.978	(0.697–1.373)	(0.900)
Toilet facility	Unimproved *	-	-	-
	Improved	1.037	(0.747–1.440)	(0.825)
Socioeconomic status	Poor *	-	-	-
	Middle	1.077	(0.739–1.570)	(0.698)
	Rich	0.927	(0.612–1.403)	(0.720)
Child Characteristics				
Milk consumption	No *	-	-	-
	Yes	1.244	(0.730–2.120)	(0.422)
Initiation of breastfeeding	Immediately *	-	-	-
	Within 1st hour	0.803	(0.555–1.161)	(0.245)
	Within 1st day	0.568	(0.468–1.164)	(0.366)
Birth order number	1st born *	-	-	-
	2nd–4th	0.611	(0.391–0.945)	**(0.030)**
	>5	0.783	(0.431–1.422)	(0.422)
Sex of child	Male *	-	-	-
	Female	0.708	(0.542–0.924)	**(0.011)**
Size of child at birth	Small *	-	-	-
	Average	0.946	(0.703–1.272)	(0.715)
	Large	0.270	(0.162–0.695)	**(0.004)**
Baby postnatal checkup within 2 months	No *	-	-	-
	Yes	1.070	(0.806–1.420)	(0.638)
Child’s age in months	0–6 *	-	-	-
	7–12	1.267	(0.869–1.848)	(0.218)
	13–24	0.474	(0.237–0.827)	**(<0.001)**
	25–36	0.779	(0.589–1.114)	(0.434)
	37–48	1.357	(0.987–1.514)	(0.378)
	49–60	1.124	(0.746–1.865)	(0.577)
Parental Characteristics				
Breastfed ever	No *	-	-	-
	Yes	0.752	(0.525–1.077)	(0.121)
Maternal smoking	No *	-	-	-
	Yes	0.767	(0.688–1.201)	(0.356)
Intake of iron during Pregnancy	No *	-	-	-
	Yes	0.921	(0.646–1.312)	(0.649)
Maternal age	15–19 *	-	-	-
	20–24	1.368	(0.744–2.515)	(0.312)
	25–29	0.599	(0.215–0.737)	**(0.045)**
	30–34	1.727	(0.834–2.578)	(0.141)
	35–39	1.915)	(0.876–2.185)	(0.103)
	40–44	1.924	(0.807–4.483)	(0.140)
	45–49	1.317	(0.350–1.951)	(0.41)
Mother’s education	No education *	-	-	-
	Primary	0.664	(0.459–0.960)	**(0.030)**
	Secondary	0.604	(0.377–0.965)	**(0.035)**
	Higher	0.787	(0.579–1.235)	(0.346)
Mother’s occupation	Not working *	-	-	-
	Working	1.110	(0.842–1.465)	(0.457)
Father’s education	No education *	-	-	-
	Primary	1.261	(0.841–1.890)	(0.261)
	Secondary	1.157	(0.705–1.898)	(0.563)
	Higher	0.829	(0.283–1.426)	(0.732)

Note: AOR = adjusted odd ratio, C.I = confidence interval, bolded = significant *p*-values, * shows reference category.

## Data Availability

The dataset used in the study was taken from the Demographic and Health Survey (DHS) website, and the files are available, accessed on 4 July 2025, at the following url: https://dhsprogram.com/data/dataset/Tanzania_Standard-DHS_2022.cfm?flag=1.

## Abbreviations

The following abbreviations are used in this manuscript:

DBM	Double-burden malnutrition
CIAF	Composite Index Anthropometric Failure
TDHS	Tanzania Demographic and Health survey
BMI	Body Mass Index
